# Study of the Layer-Type BST Thin Film with X-ray Diffraction and X-ray Photoelectron Spectroscopy

**DOI:** 10.3390/ma15020578

**Published:** 2022-01-13

**Authors:** Agata Lisińska-Czekaj, Dionizy Czekaj

**Affiliations:** Faculty of Mechanical Engineering and Ship Technology, Gdańsk University of Technology, 11/12, Narutowicza Str., 80-233 Gdańsk, Poland; dionizy.czekaj@pg.edu.pl

**Keywords:** barium strontium titanate, thin films, sol-gel method, crystal structure, X-ray photoelectron spectroscopy

## Abstract

In the present paper, results of X-ray photoelectron studies of electroceramic thin films of barium strontium titanate, Ba_1−x_Sr_x_TiO_3_ (BST), composition deposited on stainless-steel substrates are presented. The thin films were prepared by the sol-gel method. A spin-coating deposition of BST layers with different chemical compositions was utilized so the layer-type structure of (0-2) connectivity was formed. After the deposition, the thin-film samples were heated in air atmosphere at temperature *T* = 700 °C for 1 h. The surfaces of BST thin films subjected to thermal treatment were studied by X-ray diffraction. X-ray diffraction measurements confirmed the perovskite-type phase for all grown thin-film samples. The oxidation states of the elements were examined by the X-ray photoelectron spectroscopy method. X-ray photoelectron spectroscopy survey spectra as well as high-resolution spectra (photo-peaks) of the main metallic elements, such as Ti, Ba, and Sr, were compared for the layer-type structures, differing in the deposition sequence of the barium strontium titanate layers constituting the BST thin film.

## 1. Introduction

It is commonly known [1] that strontium titanate (SrTiO_3_) and barium titanate (BaTiO_3_) are known to form a solid solution (Ba_1−x_Sr_x_TiO_3_—BST) over their entire solubility range. Barium strontium titanate adopts the perovskite-type (ABO_3_) structure. It is worth noting that the term “perovskite” comes from the name of the Russian nobleman Count L.A. von Perovskiy, and is also the name of the isostructural mineral CaTiO_3_ [2]. The idealized ABO_3_ structure is cubic. For the ABO_3_-type structure, the A^2+^ ions are positioned at the elementary unit cell corners. The Ti^4+^ ions lie at the center of the unit cell and they are enclosed by a regular octahedron of O^2−^ ions. Such TiO_6_ octahedra are the origin of many significant physical properties of perovskites, such as, e.g., the ferroelectric response to external fields. This is due to the electron configuration of Ti cations, which themselves are modified by six oxygen anions surrounding the titanium. Therefore, for a discussion of the chemical and physical properties of perovskites, it is convenient to think of the structure as built-up from an array of corner-sharing TiO_6_ octahedra [2].

By adjusting the barium-to-strontium ratio, one can control the electric, dielectric, ferroelectric, and piezoelectric properties of this material in a wide range, increasing its application possibilities. It should be mentioned that substitution in the *A* sublattice for Ba^2+^, an isovalent Sr^2+^ ion with a smaller ionic radius (r(Ba^2+^) = 0.134 nm, r(Sr^2+^) = 0.112 nm) and identical configuration of the valence electron shell (Ba^2+^: 6s^2^, Sr^2+^: 5s^2^), causes a change in the mean parameter of the unit cell (<*a*>) and spontaneous deformation (*c*_0_/*a*_0_), and decreasing of the Curie temperature (*T*_C_). In the case of an increase in Sr^2+^ concentration by 1% at, *T*_C_ decreases by 4 K, the phase transition temperature *T*_p1_ (*P4mm*↔*Amm2*) decreases by 2 K, while *T*_p2_ (*Amm2*↔*R3m*) decreases by (0–1) K. The Curie temperature (*T*_C_) depends to a small extent on the quality of the substrates and the technological conditions used for synthesis of BST solid solutions, while it is determined primarily by the ratio of the Ba^2+^ and Sr^2+^ ions’ concentration. The value of the Curie–Weiss constant (*C*) also decreases. As a result, if for *x* = 0, *T*_C_ = 393 K, and *C* = 1.15 × 10^5^ K, then for *x* = 1, *T*_C_ = 29 K, while *C* = 0.67 × 10^5^ K [1]. For (Ba/Sr) ≈ 0.73/0.27, the Curie temperature of the BST solid solution can be around the room temperature [3,4].

Among the methods developed for BST thin films’ growth, one can mention radiofrequency (RF) magnetron sputtering [5,6], laser ablation deposition (PLAD) [7,8], and chemical vapor deposition (MOCVD). As well as vapor deposition techniques, chemical solution deposition (CSD) has also been utilized [9,10]. The most frequently used solution preparation approaches are metal organic decomposition (MOD) and the sol-gel method [11]. In the field of fabrication of organo-inorganic perovskite-type structure thin film, mention should be made of the method that was developed for organo-lead halide perovskite (CH_3_NH_3_PbI_3_) thin films’ fabrication [12]. In this method, the RF sputtering is used for lead-sulfide (PbS) thin films’ growth, and subsequently, the chemical treatment of PbS films in the monomethylammonium iodide (CH_6_IN) solution is performed.

A necessary step used in many areas of modern advanced technology is the deposition of thin films of electroceramic materials on various substrates [13,14]. Attempts of BST thin-film growth on single-crystal silicon substrates, platinum-coated silicon, Pt/Ti/SiO_2_/Si substrates, or perovskite-structure substrates have already been performed [15,16]. However, due to their brittleness, silicon and glass substrates do not meet the strength requirements for materials used in electromechanical devices such as actuators or energy harvesting devices, and can cause failures. On the other hand, high fracture toughness is frequently exhibited by metal substrates, even for less than 50 μm in thickness [17]. Therefore, taking into account the requirements of practical industrial application, it seems very advisable to develop a technology for the growth of efficient thin ferroelectric films on metallic substrates [18,19]. The advantage of low cost of the stainless-steel substrates made it possible to build the thin-film tunable microwave devices of the MEMS structure [20,21]. It is commonly known that metals and their alloys are characterized by high resistance to environmental conditions, they are highly technological (e.g., they ensure easy fabrication and processing), and they also provide wide possibilities of control and repair of structural components made of them. Therefore, the possibility of integrating (or built-in) a functionality into structural materials, which are primarily metals and their alloys, will create the possibility of real-time health monitoring of the structural components (of constructions and devices) and extend the maintenance cycle of components. Moreover, it will create the possibility of developing new structural functional systems [22].

Thin films of electroceramic materials differ in their properties from single crystals or bulk ceramics with the same chemical composition, e.g., see [23,24]. These differences may be caused by the state of significant stress (ranging from MPa to GPa [25]) occurring in the plane of the growth of the layers. These stresses can significantly influence the stability of the ferroelectric phase, as well as the easiness of changing the orientation of polarization in some directions.

A novelty in our experiments is using metal substrates as well as adopting the idea of graded functional materials [26,27,28,29] fabricated as the layer-type structure of (0-2) connectivity [30]. Connectivity is a critical parameter in composite materials intended for use as piezoelectric transducers, pyroelectric detectors, or as tunable microwave devices. A great advantage of such an inhomogeneous system is that it may help to make the dielectric properties better. Another great attribute is that such physical properties of ferroelectric thin films, such as strain-enhanced ferroelectric order, the Curie temperature, as well as inducing room temperature ferroelectricity in non-ferroelectric materials, can be adjusted by controlling the deformation state in thin films [31,32].

The use of organic precursors to produce barium strontium titanate thin films with improved properties is widespread. However, this type of process may leave residual carbon in thin films even after the thermal treatment step is performed to achieve crystallization [33].

The present research focuses on X-ray photoelectron spectroscopy (XPS) studies of barium strontium titanate (Ba_1−x_Sr_x_TiO_3_ or BST) electroceramic thin films. A distinctive feature of the BST thin films under study is the layer-type structure of (0-2) connectivity. By means of constitutive processes (gradation) followed by thermal processing (conventional furnace annealing), the chemical composition gradient was created in the direction perpendicular to the stainless-steel substrate. Morphology studies (Atomic Force Microscopy), crystal structure, and phase composition (X-ray diffraction method) were also utilized for thin-film characterization.

## 2. Materials and Methods

For the Ba_1−x_Sr_x_TiO_3_ thin films’ fabrication, a solution of barium acetate (Ba(CH_3_COO)_2_, Sigma-Aldrich, Darmstadt, Germany, 99%), strontium acetate (Sr(CH_3_COO)_2_, Sigma-Aldrich, 99%), and tetra-butyl titanate (Ti(OC_4_H_9_)_4_, Sigma-Aldrich, 97%) was thoroughly prepared. The precursor solution was deposited by spin-coating on stainless-steel substrates.

To form a layer-type structure of (0-2) connectivity, first, the layers with Sr mole fraction *x* = 0.5 were deposited on the stainless-steel substrate. After that, the layers with Sr mole fraction *x* = 0.4 were deposited, and later on, the ones with Sr mole fraction *x* = 0.3 were spin-coated on the top of the structure. Thus, the BST films with an “upward” gradient of the chemical composition were formed. Depositing successive layers with Sr mole fraction, *x,* ranging from *x* = 0.3 to *x* = 0.5, the BST films with a “downward” gradient were constituted. The coating process was repeated up to 30 times, resulting in the film thickness of 600 nm.

The as-deposited BST thin films were subjected to thermal treatment in an ambient atmosphere at temperature *T* = 700 ℃ for time t = 1 h (the heating rate was 1 ℃/min) to achieve crystallization.

The X-ray diffraction method (Philips PW 3710 X-ray diffractometer, *Θ*–2*Θ* mode, CoKα radiation, detector scan step size, Δ2*Θ* = 0.01°, scan type continuous, scan step time, *t* = 7 s) was utilized for investigation of the crystal structure of the thin films. Phase and structural analysis of the X-ray diffraction patterns recorded at room temperature was performed with the help of the X’pert HighScore Plus (PANalytical B.V) computer program. The available databases included the inorganic crystal structure database (ICSD) [34], the Powder Diffraction File (PDF) database by the International Centre for Diffraction Data (ICDD) [35], and the Crystallography Open Database (COD) [36]. Refinement of the structural parameters of BST thin films was performed with the Rietveld method, e.g., see [37].

Morphological investigations were performed by Atomic Force Microscopy (AFM) by using Ntegra equipment (NT-MDT [38]). Additionally, visualization and calculations were performed with a program module for processing and analysis of SPM images and SPM data by NT-MDT [39].

The PHI5700/660 Multifunctional Electron Spectrometer from Physical Electronics was used for XPS measurements. The X-ray tube had an aluminum anode, quartz monochromator, AlKα, with energy of 1486 eV. The survey spectrum (SURV) was acquired at the following conditions: pass energy 187.85, step 0.800 eV, time 20 ms, range from −2 to 1400 eV. Conditions for acquiring the high-resolution spectrum (HRES) were as follows: pass energy 23.50, step 0.100 eV, time 100 ms. The XPS method provides information on chemical composition and chemical states of atoms in a surface layer comprising several atomic layers.

## 3. Results and Discussion

### 3.1. Crystal Structure of BST Ceramic Thin Film

**Figure 1:**
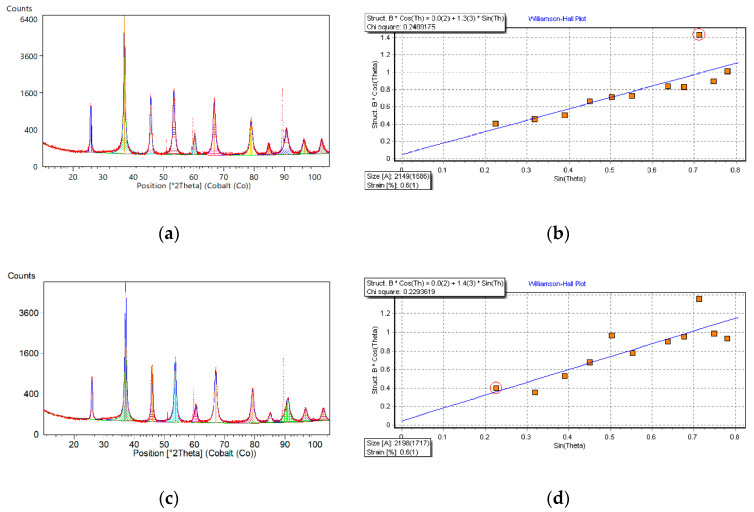
(**a**) X-ray diffraction pattern of the “upgraded” (*x* = 5-4-3) sol-gel deposited BST thin film on the stainless-steel substrate. (**b**) Williamson-Hall plot of the “upgraded” (*x* = 5-4-3) BST thin film. (**c**) X-ray diffraction pattern of the “downgraded” (*x* = 3-4-5) BST thin film on the stainless-steel substrate. (**d**) Williamson-Hall plot of the “downgraded” (*x* = 3-4-5) BST thin film.

As an example, X-ray diffraction patterns of the BST ceramic thin films deposited by the sol-gel method on stainless-steel substrates and fired (crystallized) at *T* = 700 °C for 1 h are shown in Figure 1 (dots). The visual inspection of the diffraction data has shown that three diffraction lines, namely, 2*Θ* = 51.02°, 2*Θ* = 59.65°, and 2*Θ* = 89.41°, are due to the stainless-steel substrate (Figure 1a,c, dotted line).

The line profile analysis was performed after raw data processing and the results of calculations are also presented in Figure 1a,c (solid line). The Williamson-Hall plots are shown in Figure 1b,d for so-called “upgraded” (*x* = 5-4-3, the first significant digits of the Sr mole fraction were used for abbreviation) and “downgraded” (*x* = 3-4-5) BST thin films, respectively. One can see from the Williamson-Hall plots that the average crystallite size is <*D*> = 215 nm for “upgraded” BST film, whereas for “downgraded” BST thin film, <*D*> = 220 nm. The average strain (<*ε*>), which is a measure of micro-deformations (Δ*d*_hkl_/*d*_hkl_, where *d*_hkl_ is an interplanar distance) equals to <*ε*> = 0.6% for both thin-film-layered structures.

**Figure 2:**
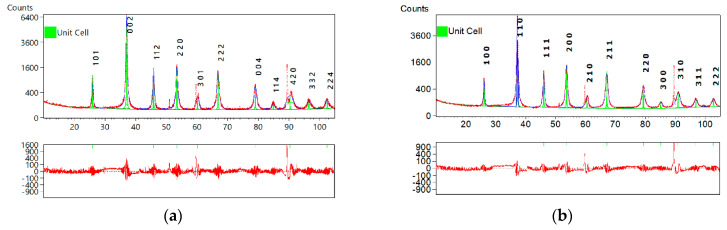
Results of searching the unit cell for (**a**) “upgraded” (*x* = 5-4-3) and (**b**) “downgraded” (*x* = 3-4-5) BST thin film deposited by the sol-gel method on the stainless-steel substrate and fired at *T* = 700 °C.

The unit cell search calculations were performed on the basis of the X-ray diffraction patterns. Once possible unit cells were found, the next step was to refine a selected unit cell and to decide on a lattice and possible space group. The results are presented in Figure 2, and Table 1 and Table 2.

It was found that the “upgraded” (*x* = 5-4-3) BST thin film exhibits an orthorhombic structure with a space group *Imma* (74) (Figure 2a, Table 1), whereas the crystal structure of the “downgraded” (*x* = 3-4-5) BST thin film is well-described by a cubic symmetry with a space group *P23* (195) (Figure 2b, Table 2).

One can see from Table 1 and Table 2 that the global parameters of the fitting process confirm the good quality of the obtained results, with the value of the GOF (goodness-of-fit) parameter ranging from 1.71 to 1.88. It is worth noting that the bottom panels in Figure 2 show the difference between the intensities of the observed and calculated patterns. One can see that the substantial value of the difference appeared only at the diffraction angles where the stainless-steel substrate manifested itself.

**Figure 3:**
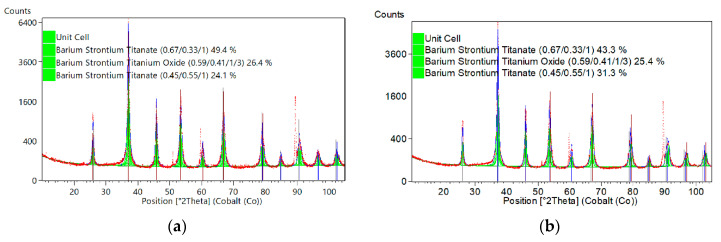
Results of the Rietveld analysis of X-ray diffraction patterns of the (**a**) “upgraded” (*x* = 5-4-3) BST thin film and (**b**) “downgraded” (*x* = 3-4-5) BST thin film.

Detailed structural analysis of the X-ray diffraction patterns was performed with the Rietveld refinement method. The Rietveld method was used to determine precise lattice constants from a measurement. Resulting diffraction patterns are presented in Figure 3 and results of the calculations are provided in Table 3 and Table 4.

As a model structure of BST films with an “upward” (Sr molar fraction: *x* = 0.5, *x* = 0.4, and *x* = 0.3) and “downward” gradient of the chemical composition, the following powder diffraction patterns were taken as initial structures for structural parameters’ refinement:

- Barium Strontium Titanate, chemical formula: Ba_0_._67_O_3_Sr_0_._33_Ti_1_-tetragonal, space group: *P4mm*(99), ICSD collection code: 54150, PDF number (calculated powder diffraction data) 01-089-0274.
- Barium Strontium Titanium Oxide, chemical formula: Ba_0_._592_O_3_Sr_0_._408_Ti_1_-cubic, space group: *Pm-3m*(221), ICSD collection code: 90006, PDF number (calculated powder diffraction data): 01-070-3628.
- Barium Strontium Titanate, chemical formula: Ba_0_._45_O_3_Sr_0_._55_Ti_1_-cubic, space group: *Pm-3m*(221), ICSD collection code: 154403, PDF code: 00-039-1395.

Results of the calculations were performed for the XRD profile modeled with a Pseudo-Voigt function. Global parameters of the Rietveld fitting are provided in Table 3.

One can see from Table 3 that the global parameters of the fitting process performed for XRD diffraction patterns of the thin films with both configurations of the layers, i.e., structures with upward and downward chemical composition gradient, exhibited low values of *R*-parameters, typical for thin films deposited on stainless-steel [40].

The relevant parameters of BST phases used as initial structures for structural parameters’ refinement of “upgraded” (*x* = 5-4-3) and “downgraded” (*x* = 3-4-5) BST thin films are presented in Table 4.

One can see from Table 4 that the weight fraction of the phases constituting the BST thin films and retrieved on the basis of the Rietveld refinement of the crystal structure consists mainly of the tetragonal phase, namely, 49.4% for “upgraded” (*x* = 5-4-3) and 43.3% for “downgraded” (*x* = 3-4-5) BST thin film. The weight fraction of the “middle layer” (i.e., for *x* = 0.4) of the three-layer-type structure of (0-2) connectivity differs slightly—26.4% for “upgraded” and 25.4% for the “downgraded” structure. Calculated density for all phases is higher in the case of the “downgraded” (*x* = 3-4-5) BST thin film.

### 3.2. Morphology Studies of the Thin Film

**Figure 4:**
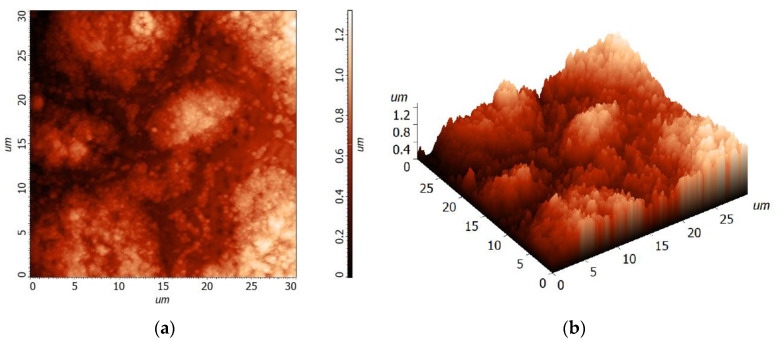
(**a**) 2D topography and (**b**) 3D topography of the “upgraded” (*x* = 5-4-3) BST thin film deposited on the stainless-steel substrate by the sol-gel method, followed by spin-coating deposition.

**Figure 5:**
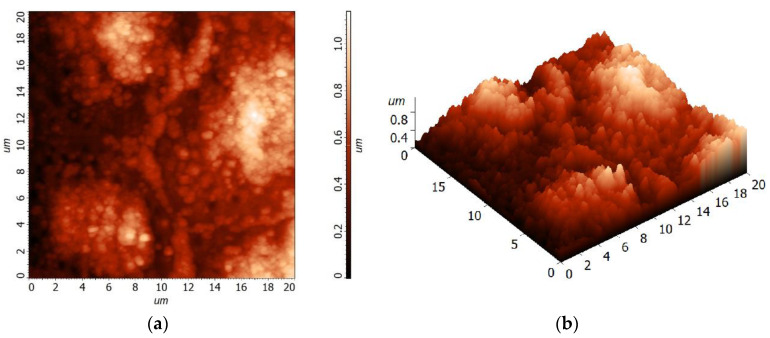
(**a**) 2D topography and (**b**) 3D topography of the “downgraded” (*x* = 3-4-5) BST thin film deposited on the stainless-steel substrate by the sol-gel method, followed by spin-coating deposition.

The microstructure of the BST thin films was studied by Atomic Force Microscopy (AFM). The morphology of the “upgraded” (*x* = 5-4-3) BST thin-film surface of 30 × 30 μm is shown in Figure 4, whereas the morphology of the “downgraded” (*x* = 3-4-5) BST thin film is shown in Figure 5.

Statistical analysis of the surface roughness of the “upgraded” (*x* = 5-4-3) BST thin film deposited on the stainless-steel substrate has shown that the average roughness of the thin-film’s surface is Sa = 185.31 nm and root mean square, Sq = 232.024 nm.

On the other hand, the surface roughness of the “downgraded” (*x* = 3-4-5) BST thin film deposited on the stainless-steel substrate exhibited the average roughness of the thin-film’s surface equal to Sa = 166.997 nm and root mean square, Sq = 207.321 nm.

It is worth noting that the layer-type structure (i.e., “upgraded” (*x* = 5-4-3) and “downgraded” (*x* = 3-4-5)) BST thin films were deposited on polished stainless-steel of AISI-304–type, which was characterized with an arithmetic mean height of the surface, Sa = 0.05 μm [41]. Therefore, one can conclude that the BST thin-film surface roughness was caused by conditions of the thin-film growth rather than the roughness of the substrate.

To perform the grain size analysis, the source data AFM images shown in Figure 4 and Figure 5 were processed with the help of the Image Analysis P9 program by NT-MDT [42]. First, the sharpening filter was utilized, and then the procedure of grain identification with a watershed transformation was performed. Thus, the obtained picture with clearly visible grains and grain boundaries was statistically analyzed. It was found that statistical distribution of the thin-film grain sizes depended on the synthesis conditions [43]. It can be seen from the obtained data (Table 5 and Table 6) that the “downgraded” (*x* = 3-4-5) BST thin films deposited on stainless-steel exhibited smaller values of calculated average geometric parameters by about 24–28%, namely, area, average size (26%), length (25%), mean width (28%), volume, perimeter (24%), and diameter (26%).

**Figure 6:**
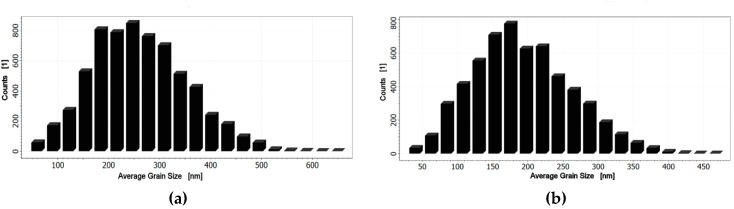
Histograms of the average grain size of (**a**) “upgraded” (*x* = 5-4-3) and (**b**) “downgraded” (*x* = 3-4-5) BST thin films deposited on the stainless-steel substrate.

As an example, histograms of the distribution of average size are shown in Figure 6.

### 3.3. X-ray Photoelectron Studies of Graded BST Thin Films

**Figure 7:**
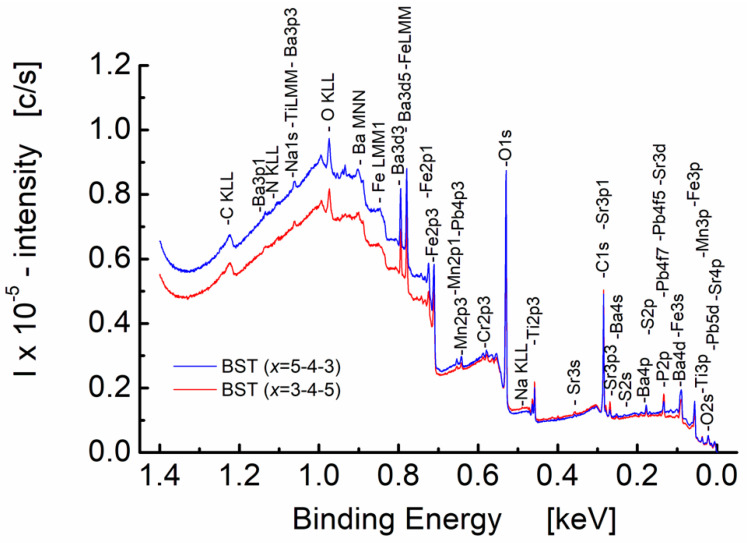
X-ray photoelectron survey spectra recorded from the surface of BST thin films deposited on the stainless-steel substrate and fired at *T* = 700 °C. BST (*x* = 5-4-3)—blue line, and BST (*x* = 3-4-5)—red line.

X-ray photoelectron survey spectra for sol-gel-derived BST thin films are shown in Figure 7.

One can see from Figure 7 that intensities of the X-ray photoelectron survey spectra, collected from the surface layer comprising several atomic layers, depend on the deposition sequence of the barium strontium titanate layers constituting the BST thin film. The intensity of the spectrum for the layer-type structures with the Ba_0_._7_Sr_0_._3_TiO_3_ layer on the top of the layered structure (i.e., BST with *x* = 5-4-3-type structure, blue line in Figure 7) is higher than for the case when the Ba_0_._5_Sr_0_._5_TiO_3_ layer is on the top of the structure (i.e., BST with *x* = 3-4-5-type structure, red line in Figure 6) for the binding energy value within the range *E*_B_ = 1.4–0.7 keV. On the contrary, for the lower values of the binding energy (i.e., smaller than 0.7 keV), the difference in intensities is less visible.

In principle, the peak positions in terms of binding energy provide information about the chemical state for a material. The data in Figure 7 provide evidence for the following series of peaks corresponding to photoemissions from the different core shells: *s*, *p*, and *d* for barium (Ba 3p1, Ba 3p3, Ba 3d3, Ba 3d5, Ba 4s, Ba 4p, Ba 4d), strontium (Sr 3s, Sr 3p1, Sr3 p3, Sr3 d), and titanium (Ti 3p).

It is commonly known, e.g., see [44,45], that the best way to compare XPS intensities is via percentage atomic concentrations. The atomic concentrations of the three main metals, namely titanium, strontium, and barium, calculated from the survey spectra (Figure 7) of the BST thin films, are presented in Table 7.

Taking into consideration the chemical composition of the upper layer of the BST thin film with the sequence of layers *x* = 3-4-5 (i.e., “downgraded” BST), where the Ba_0_._5_Sr_0_._5_TiO_3_ layer is on the top of the structure, the atomic concentration (*C*) calculated on the basis of the number of moles of an element in relation to the total moles of the elements in the compound (oxygen excluded), is: *C*_Ba_ = 25 at.%, *C*_Sr_ = 25 at.%, and *C*_Ti_ = 50 at.%. For the BST thin film exhibiting the sequence of layers *x* = 5-4-3 (“upgraded” BST), where the Ba_0_._7_Sr_0_._3_TiO_3_ layer is on the top of the layered structure, the calculated atom percent (at.%) is: *C*_Ba_ = 35 at.%, *C*_Sr_ = 15 at.%, and *C*_Ti_ = 50 at.%.

**Figure 8:**
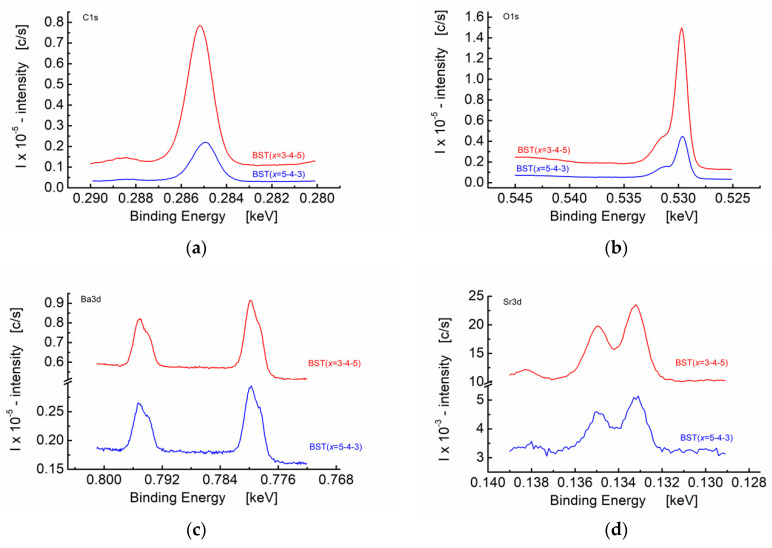

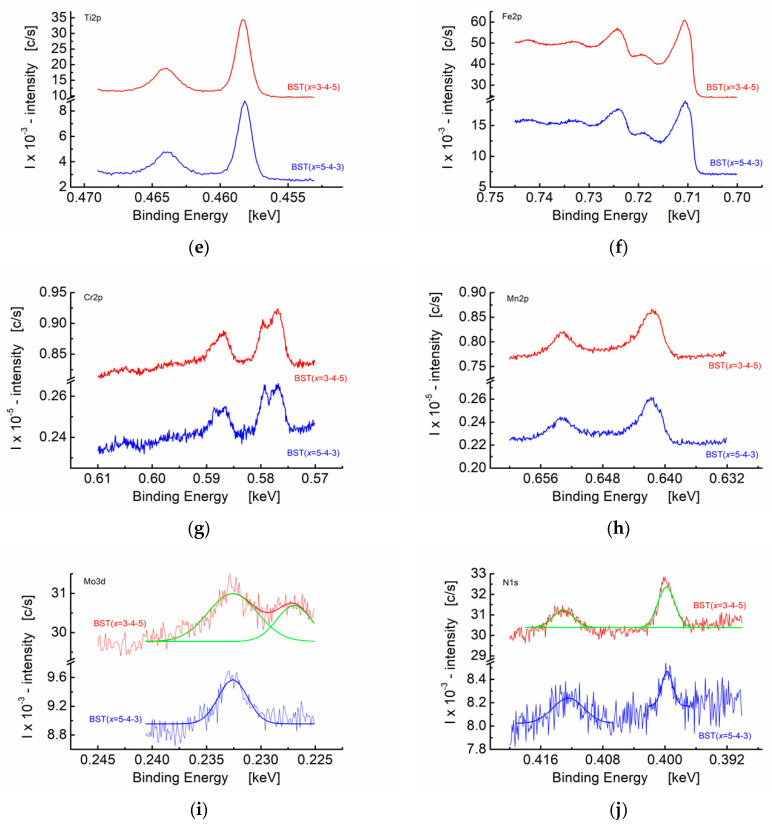
X-ray photoelectron high-resolution spectra for BST thin films differing in the sequence of deposition of the barium strontium titanate layers constituting the thin film. BST (*x* = 5-4-3)—blue line, and BST (*x* = 3-4-5)—red line. (**a**) C 1s, (**b**) O 1s, (**c**) Ba 3d, (**d**) Sr 3d, (**e**) Ti 2p, (**f**) Fe 2p, (**g**) Cr 2p, (**h**) Mn 2p, (**i**) Mo 3d, and (**j**) N 1s.

High-resolution X-ray photoelectron spectra for BST thin films differing in the sequence of the deposition of the barium strontium titanate layers constituting the thin film are shown in Figure 8.

From a visual inspection of the spectra shown in Figure 8, one can see smooth and monotonic curves for C 1s, O 1s, Ba 3d, Sr 3d, Ti 2p, and Fe 2p photoelectron lines. However, it can be seen that intensities of the relevant photo-peaks recorded under the same measuring conditions are higher for BST (*x* = 3-4-5) thin films as compared to BST (*x* = 5-4-3). The most intense photoelectron lines are usually symmetrical in shape and exhibit the smallest width (they are usually the narrowest lines) in the observed spectrum [44].

The binding energy of the core electrons basically depends on the elements to which it is bonded. The observed “chemical shift” is due to the charge transfer. Therefore, the bonding environment (chemical state) can be established. Chemical shifts can arise due to several reasons, e.g., variation in electronegativity, molecular environment, positions in the lattice, oxidation states, etc. Figure 8a illustrates the chemical shift effect of C 1s in the BST thin film. The main line at binding energy c.a. *E*_B_ = 285 eV can be ascribed to C-C and C-H bonds. The weaker line at *E*_B_ = 288.5 eV can be ascribed to C-O and C=O bonds. The core level binding energy of the carbon atom increases as the electronegativity of the neighboring atoms, in this case oxygen, increases, resulting in the chemical shift. There is also a difference in the peak positions of the main line at the C 1s binding energy spectrum between the BST (*x* = 5-4-3) and BST (*x* = 3-4-5) (blue and red line, respectively, Figure 8a). The Gauss approximation yields *E*_B_ = 285.19 eV and *E*_B_ = 284.98 eV, for BST (*x* = 5-4-3) and BST (*x* = 3-4-5) thin-film structures, respectively.

Figure 8b illustrates the O 1s photoelectron lines’ spectrum. The main line at c.a *E*_B_ = 530 eV can be ascribed to possible oxides, whereas the weaker line at *E*_B_ = 531.7 eV provides information on possible surface impurities, likely H_2_O and OH [46].

One can see from Figure 8c that the Ba 3d photoelectron lines are split. The line at *E*_B_ = 778.8 eV may be ascribed to BaCrO_4_, whereas the line at *E*_B_ = 780 eV can be recognized as BaCO_3_. The photoelectron lines of Sr 3d are shown in Figure 8d. The titanium peak corresponding to photoemission from core shell *p* (Ti 2p line, shown in Figure 8e) exhibits a binding energy similar to BaTiO_3_, i.e., *E*_B_ = 458.4 eV.

Peaks corresponding to photoemissions from Fe 2p, Cr 2p, Mn 2p, and Mo 3d are shown in Figure 8f–i, respectively. It is worth noting that the stainless-steel substrate used for the thin-film growth may contain the above-mentioned elements. Therefore, the photoemission line that peaks at *E*_B_ = 711.1 eV can be assigned to oxidized iron, Fe_2_O_3_ (Figure 8f). Chromium Cr 2p photoemission lines are found to be split (Figure 8g). The line at *E*_B_ = 577 eV can be assigned to Cr_2_O_4_, whereas the line at *E*_B_ = 580 eV is likely to be Cr_2_O_3_. Wide asymmetrical lines are visible in Figure 8h. The photoemission line at *E*_B_ = 641.7 eV may be ascribed to Mn_2_O_3_, whereas the shoulder that appeared at approximately *E*_B_ = 643.5 eV could be assigned to MnO_2_. Peaks corresponding to photoemission from molybdenum are shown in Figure 8i. It can be seen that both BST (*x* = 5-4-3) and BST (*x* = 3-4-5) show the photoemission peak at *E*_B_ = 233 eV (Mo 3d3) that can be ascribed to MoO_2_. On the other hand, the Mo 3d5 photoemission line that peaks at *E*_B_ = 230 eV was visible for BST (*x* = 3-4-5) thin film. Figure 8j illustrates the N 1s photoelectron lines’ spectrum that is caused by NH_3_-containing compounds, probably.

On the basis of the XPS high-resolution spectra, the atomic concentrations were calculated and the results relevant to the three main metals, namely, Ti, Sr, and Ba, are presented in Table 8.

One can see from Table 8 that the atomic concentration of titanium is less than the theoretical one, namely, *C*_Ti_ = 50 at.% for Δ*C*_Ti_ = (2.2–1.94) at.%. Additionally, the atom percentage for barium was found to be lower than the theoretical one calculated for the upper-layer composition of the three-layered BST structure for c.a. Δ*C*_Ba_ = (3.53–1.74) at.%. On the contrary, the strontium atomic concentration was found to be higher that the calculated theoretical one for Δ*C*_Sr_ = (5.73–3.68) at.%. The obtained results are within the limits of the quantitative accuracy of the atomic percent values calculated from the major XPS peaks (90–95% for each peak). One can also conclude that the differences in the atomic concentrations indirectly proved the preservation of spatially heterogeneous chemical composition of the layers comprising the three-layer BST structure after the heat treatment of the films.

## 4. Conclusions

By means of the sol-gel method followed by thermal processing, BST electroceramic thin films exhibiting the three-layer-type structure of (0-2) connectivity were successfully fabricated. It was found that the average crystallite size was <*D*> = 215 nm for BST (*x* = 5-4-3) thin films, whereas for BST (*x* = 3-4-5) thin film, <*D*> = 220 nm. The micro-deformation expressed in terms of the average strain was <*ε*> = 0.6% for both thin-film-layered structures.

It was found that the weight fraction of the phases constituting the BST thin films consists mainly of the tetragonal phase (*P4mm*, (99)), namely, 49.4 wt.% and 43.3 wt.% for BST (*x* = 5-4-3) and BST (*x* = 3-4-5) thin films, respectively. The weight fraction of the cubic (*Pm-3m* (221)) “middle layer” (i.e., for *x* = 0.4) differed slightly—26.4 wt.% for BST (*x* = 5-4-3) and 25.4 wt.% for the BST (*x* = 3-4-5) structure. The bottom layer of BST (*x* = 5-4-3) and the upper layer of BST (*x* = 3-4-5) were found to be cubic (*Pm-3m* (221)), and their weight fractions were 24.1 wt.% and 31.3 wt.%, respectively.

Statistical analysis of the surface roughness of the BST thin films has shown that the average roughness of the thin-film’s surface was Sa = 185.31 nm and Sa = 166.997 nm for BST (*x* = 5-4-3) and BST (*x* = 3-4-5) structures, respectively. Taking into consideration that the average surface roughness of the stainless-steel substrate was Sa = 50 μm, one can conclude that the BST thin-film surface roughness was caused mainly by conditions of the thin-films’ growth rather than the roughness of the substrate.

Statistical analysis of the grain distribution made it possible to calculate average geometrical parameters of the grains of BST thin films deposited on stainless-steel. The average grain size was found to be <*D*> = 201 nm and <*D*> = 274 nm for the BST (*x* = 3-4-5) and BST (*x* = 5-4-3) layered structures, respectively. The “downgraded” (*x* = 3-4-5) BST layered structure exhibited smaller values of the calculated average geometric parameters by about 24–28%, as compared to the “upgraded” (*x* = 5-4-3) BST structure.

Results of XPS spectra were found to depend on the deposition sequence of the barium strontium titanate layers constituting the BST thin film. This is explainable if one takes into consideration that after formation of the spatially inhomogeneous three-layer BST structure of (0-2) connectivity, the as-deposited thin films were subjected to the heat treatment at *T* = 700 °C for *t* = 1 h. Therefore, the process of transformation of the deposit into the polycrystalline thin film took place, as well as the mass transfer in favor of a more uniform concentration of chemical species occurred. The differences in XPS spectra, however, taken from the surface layer comprising several atomic layers, may be an indirect proof of preserving the spatially heterogeneous chemical composition of the BST thin films.

## Figures and Tables

**Table 1:** Results of the unit cell search calculations for the “upgraded” (*x* = 5-4-3) BST thin film.

Global Parameters	Value	Relevant Parameters of Unit Cell	Value
Profile function	Pseudo-Voigt	Space group (No.)	*Imma* (74)
Background	Polynomial	Lattice parameters:	
*R* (expected), [%]:	11.05	*a*, [Å]	5.648
*R* (profile), [%]	10.01	*b*, [Å]	5.594
*R* (weighted profile), [%]	20.84	*c*, [Å]	5.620
GOF	1.885	Angle *α = β* = *γ*, [°]	90
*d*-statistic	0.10367	*V×* 10^−6^, [pm^3^]	177.5975

**Table 2:** Results of the unit cell search calculations for the “downgraded” (*x* = 3-4-5) BST thin film.

Global Parameters	Value	Relevant Parameters of Unit Cell	Value
Profile function	Pseudo-Voigt	Space group (No.)	*P23* (195)
Background	Polynomial	Lattice parameters:	
*R* (expected), [%]:	11.86	*a*, [Å]	3.966
*R* (profile), [%]	8.49	*b*, [Å]	3.966
*R* (weighted profile), [%]	20.29	*c*, [Å]	3.966
GOF	1.711	Angle *α = β* = *γ* [°]	90
*d*-statistic	0.01508	*V×* 10^−6^*,* [pm^3^]	62.37806

**Table 3:** Global parameters of the Rietveld method fitting used for structural parameters’ refinement for “up-” and “down-graded” BST thin films deposited on stainless-steel.

Global Parameters	Value (*x* = 5-4-3) BST	Value (*x* = 3-4-5) BST
Profile function	Pseudo-Voigt	Pseudo-Voigt
Background	Polynomial	Polynomial
*R* (expected), [%]:	11.07193	11.88390
*R* (profile), [%]	13.69155	13.47152
*R* (weighted profile), [%]	24.94008	23.60903
GOF	5.07398	3.94674
*d*-statistic	0.06714	0.01228

**Table 4:** Relevant parameters of the BST phases identified for “upgraded” (*x* = 5-4-3) (left side of the column) and “downgraded” (*x* = 3-4-5) (right side of the column) BST thin film.

Relevant Parameters	Barium Strontium Titanate (0.67/0.33/1)		Barium Strontium Titanium Oxide (0.59/0.41/1/3)		Barium Strontium Titanate (0.45/0.55/1)	
Formula sum	O_3_._00_Ti_1_._00_Sr_0_._33_Ba_0_._67_		O_3_._00_Ti_1_._00_Sr_0_._41_Ba_0_._59_		O_3_._00_Ti_1_._00_Sr_0_._55_Ba_0_._45_	
Formula mass, [g/mol]	216.8239		212.9465		205.2415	
Density (*calc*.), [g/cm^3^]	5.7490	5.7679	5.5852	5.5961	5.5124	5.5191
Weight fraction, [%]	49.4	43.3	26.4	25.4	24.1	31.3
Space group (No.)	P 4 m m (99)		P m-3 m (221)		P m-3 m (221)	
Lattice parameters:						
*a*, [Å]	3.9754(2)	3.9707(2)	3.9854(4)	3.9828(4)	3.9540(4)	3.9524(2)
*b*, [Å]	3.9754(2)	3.9707(2)	3.9854(4)	3.9828(4)	3.9540(4)	3.9524(2)
*c*, [Å]	3.9623(4)	3.9587(5)	3.9854(4)	3.9828(4)	3.9540(4)	3.9524(2)
Angle *α* = *β* = *γ*, [°]	90		90		90	
*V×* 10^−6^, [pm^3^]	62.61871	62.41287	63.30238	63.17865	61.81735	61.74294

**Table 5:** Parameters of the grain analysis of the “upgraded” (*x* = 5-4-3) BST thin films deposited on stainless-steel substrates.

*x* = 5-4-3 BST Film	Area	Average Size	Length	Mean Width	Aspect Ratio	Volume	Perimeter	Diameter
Unit	μm^2^	μm	μm	μm	[1]	μm^3^	μm	μm
Average	0.0834	0.274	0.425	0.178	2.411	4.952	1.037	0.309
SD	0.0534	0.0907	0.154	0.0597	0.592	4.876	0.488	0.102

**Table 6:** Parameters of the grain analysis of the “downgraded” (*x* = 3-4-5) BST thin films deposited on stainless-steel substrates.

*x* = 3-4-5 BST Film	Area	Average Size	Length	Mean Width	Aspect Ratio	Volume	Perimeter	Diameter
Unit	μm^2^	μm	μm	μm	[1]	μm^3^	μm	μm
Average	0.0455	0.201	0.316	0.130	2.463	1.590	0.787	0.227
SD	0.0304	0.0698	0.120	0.0454	0.647	1.775	0.376	0.0787

**Table 7:** Atomic concentration table according to the results of the XPS survey spectrum measurement.

Element/Value	Ti 2p	Sr 3p3	Ba 3d3
BST (*x* = 3-4-5), [at.%]	48.06	31.38	20.56
BST (*x* = 5-4-3), [at.%]	16.43	72.31	11.26

**Table 8:** Atomic concentration table according to the results of the XPS spectrum measurement (HRES).

Element/Value	Ti 2p	Sr 3d	Ba 3d3
BST (*x* = 3-4-5), [at.%]	47.80	30.73	21.47
BST (*x* = 5-4-3), [at.%]	48.06	18.68	33.26

## Data Availability

The data presented in this study are available on request from the corresponding author.
